# Vitamin D Deficiency: A Potential Modifiable Risk Factor for Cardiovascular Disease in Children with Severe Obesity

**DOI:** 10.3390/children4090080

**Published:** 2017-08-28

**Authors:** Anoop Mohamed Iqbal, Amanda R. Dahl, Aida Lteif, Seema Kumar

**Affiliations:** 1 Division of Pediatric Endocrinology, Department of Pediatric and Adolescent Medicine, Mayo Clinic, 200 First Street SW, Rochester, MN 55905, USA; iqbal.anoop@mayo.edu (A.M.I.); lteif.aida@mayo.edu (A.L.); 2 Department of Pediatric and Department of Pediatric and Adolescent Medicine, Mayo Clinic, Rochester, MN 55905, USA; dahl.amanda2@mayo.edu

**Keywords:** vitamin D, dyslipidemia, high-density lipoprotein cholesterol, childhood obesity, severe obesity

## Abstract

Severe obesity is associated with abnormal lipids and increased risk for cardiovascular disease. Obesity is a risk factor for vitamin D deficiency. We examined relationship between 25-hydroxy vitamin D (25(OH)D) concentrations and lipids in children with severe obesity. Medical records of 376 children were reviewed. Linear regression models and logistic regression were used to examine the relationship between 25(OH)D and lipids after adjustment for age, gender, season of blood draw, body mass index (BMI) z-score, and BMI % of 95th percentile. Two-hundred sixty-three out of 376 children (70%) had 25(OH)D concentrations < 30 ng/mL. Concentrations of 25(OH)D were positively correlated with those of high-density lipoprotein cholesterol (HDL-C) (*r^2^* = 0.08, *r* = 0.22, *β* = 0.16, 95% CI = 0.05–0.27, *p* = 0.004). HDL-C was lower in children with 25(OH)D < 30 ng/mL (*n* = 263) compared to those with 25(OH)D ≥ 30 ng/mL (*n* = 113) (41.3 ± 10.2 vs. 46.4 ± 12 mg/dL, *p* < 0.0001). Children with 25(OH)D concentrations < 30 ng/mL had greater adjusted odds of low HDL-C (<40 mg/dL) compared with those with 25(OH)D ≥ 30 ng/mL (47.9% vs. 29.2%, OR 2.15 (1.33–3.51), *p* = 0.0019). Total cholesterol and non-HDL-C were not correlated with 25(OH)D concentrations. Vitamin D deficiency is highly prevalent in children with severe obesity. Prospective clinical trials are warranted to determine if vitamin D supplementation can improve HDL-C and potentially decrease risk for cardiovascular disease in children with obesity.

## 1. Introduction

Childhood obesity has emerged as an important public health problem in the United States and in other countries [1,2,3]. Severe obesity is defined as having a body mass index (BMI) ≥ 120% of 95th percentile for age and gender, or BMI > 35 kg/m^2^, whichever is lower, and affects 4–6% of youth in the United States [1,4]. Although the prevalence of obesity appears to have plateaued among children, the prevalence of severe obesity is increasing [1,4,5]. 

Childhood obesity is associated with several cardiometabolic risk factors including an adverse lipid profile [6,7,8]. A significant proportion of children with severe obesity continue to be obese as adults and therefore to be at high risk for cardiovascular disease. The prevalence and severity of dyslipidemia increase with the degree of obesity [4,8]. In children, adolescents, and young adults, autopsy examination of coronary arteries and aorta show early vascular changes, suggesting that atherosclerosis begins in childhood [9,10,11]. The atherosclerotic lesions are positively associated with serum levels of total cholesterol and non-high-density lipoprotein cholesterol and are negatively associated with levels of high-density lipoprotein (HDL) cholesterol [11]. In children with severe obesity, modifiable risk factors for dyslipidemia should be promptly identified. 

Obesity is a known risk factor for vitamin D deficiency [12,13,14,15]. Circulating concentration of 25-hydroxy vitamin D (25(OH)D) is considered to be the best indicator of vitamin D nutritional status. 25(OH)D concentrations are inversely associated with the severity of obesity [16,17]. There is lack of consensus on what optimal 25(OH)D concentrations are, with some experts such as those from the Endocrine Society suggesting a minimum concentration of 30 ng/mL [18], while others such as those from the Institute of Medicine consider a concentration above 20 ng/mL as sufficient [19,20]. Two-hundred eighty-four children out of 581 (49%) from the 2003–2006 National Health and Nutrition Examination survey between the ages 6–18 years with severe obesity were noted to have 25(OH)D < 20 ng/mL, and 92% had 25(OH)D levels < 30 ng/mL [16]. Similarly, 92% of children evaluated at an obesity clinic in Texas had 25(OH)D < 30 ng/mL [17]. Mechanisms felt to be contributing to this relationship include sequestration of the fat soluble vitamin D within the adipose tissue [12], its inadequate input from exogenous sources such as dietary and cutaneous, negative feedback from higher circulating 1,25-dihydroxyvitamin D (1,25(OH)_2_D) levels in obesity and/or volumetric dilution, and increased clearance by a large body fat pool [21]. 

Besides its classical physiological function of regulation of calcium and bone metabolism, there is increasing interest in the extra-skeletal effects of vitamin D [22]. Epidemiological studies in adults have linked vitamin D deficiency with greater risk for major adverse cardiovascular events as well as higher all-cause mortality and cardiovascular mortality [23,24,25]. 25(OH)D concentrations have been noted to be positively correlated with HDL cholesterol in children [15,26,27,28,29,30,31]. The metabolic and cardiovascular implications of low vitamin D status in obese children and adolescents are not very well characterized. Data on the relationship between vitamin D status and lipid profile in children with obesity is inconsistent, with some studies suggesting an association between vitamin D status and lipids [27,32,33,34] while others have not found such relationship [35,36]. These inconsistencies are likely related to differences in study subjects with regards to age, ethnicity/race, pubertal stage, geographic location, dietary habits, physical activity, and assays for measuring 25(OH)D. 

Furthermore, there is further scarcity of information on the relationship between vitamin D deficiency and risk factors of cardiovascular disease (CVD) in children with severe obesity, the subgroup with extremely highest risk for cardiovascular disease risk factors such as dyslipidemia and vitamin D deficiency [33,35].

The objective of the study was: (1) to examine the relationship between 25(OH)D concentrations and lipids among children with severe obesity; and (2) to determine if either of the two suggested cut-off values of 25(OH)D (20 ng/mL and 30 ng/mL) is associated with greater odds of dyslipidemia in this population. The two cut-off concentrations for 25(OH)D were examined due to lack of consensus on definition of normal vitamin D status. 

## 2. Materials and Methods

The study was a retrospective chart review of the electronic medical records of children between 2 and 18 years old with severe obesity who had simultaneous measurement of 25(OH)D levels and lipids (total cholesterol, HDL cholesterol, and non-HDL cholesterol) between 1 January 2008 and 31 October 2015 at the outpatient endocrinology clinic at Mayo Clinic, Rochester, MN, USA. Severe obesity was defined as BMI ≥ 120% of 95th percentile for age and gender or BMI > 35 kg/m^2^, whichever was lower [4,37,38]. The study was approved by the Institutional Review Board of Mayo Clinic, Rochester, MN, USA (n° 16-001820). Records of only those patients who had given research authorization were reviewed. 

Subjects without BMI measurements within 1 year of collection of the laboratory tests were excluded. Height and weight were measured by trained personnel using standardized equipment. Age and sex specific BMI percentiles were determined using Center for Disease Control growth charts [39]. In patients with multiple measurements of 25(OH)D during the study period, only the first measurement was included in the analysis. Subjects with type 1 and type 2 diabetes mellitus, celiac disease, hypoparathyroidism, hyperparathyroidism, those who had undergone organ transplant, or were receiving systemic glucocorticoids or statins were excluded. 25(OH)D was measured using liquid chromatography-tandem mass spectrometry (LC-MS/MS). 

Total 25(OH)D concentration of each sample was calculated using internal standards for 25(OH)D_2_ and 25(OH)D_3_ (Mayo Medical Laboratories, Rochester, MN, USA). Total cholesterol and HDL cholesterol were measured by an enzymatic calorimetric assay (Roche Diagnostics, Indianapolis, IN, USA). Non-HDL cholesterol was calculated as total cholesterol minus HDL cholesterol. 

Abnormal lipid values were defined according to the National Heart, Lung, and Blood Institute's (NHLBI) Expert Panel on Integrated Guidelines for Cardiovascular Health and Risk Reduction in Children and Adolescents [40]. The criteria for abnormal values were: total cholesterol ≥ 200 mg/dL, HDL cholesterol < 40 mg/dL, and non-HDL cholesterol ≥ 145 mg/dL. We chose to not analyze measurements of triglycerides and LDL cholesterol as fasting prior to the blood draw could not be verified from medical record review. 

### Statistical Analysis

Continuous variables were reported as mean and standard deviation (SD), while categorical variables were summarized as frequency and percentage. A series of linear regression models were used with lipids as the outcome, and with dichotomized vitamin D as the primary predictor; and each model was adjusted for age, gender, season of blood draw, and a BMI metric (z-score or % of the 95th percentile). We chose to include BMI z-score as it is a metric that has been traditionally used for clinical and research purposes. However, BMI z-score in children with severe obesity does not correlate with adiposity [41]. Instead BMI % of the 95th percentile is now recommended as a preferred metric in children with severe obesity due to better correlation with adiposity [41]. Additionally, dichotomized lipid values were compared between vitamin D sub-groups defined using 25(OH)D cut-off concentrations of 20 ng/mL or 30 ng/mL with logistic regression, adjusting for the same characteristics as noted above. All statistical analysis was performed using JMP statistical analysis software (SAS Institute Inc., Cary, NC, USA). All statistical tests were two-sided and α set at 0.05 for statistical significance.

## 3. Results

Demographic and laboratory characteristics are outlined in Table 1. The mean age of the study subjects (*n* = 376) was 13.48 ± 3.95 years, and 208 (55%) of the subjects were females. Mean BMI was 38.32 ± 8.12 kg/m^2^, and mean BMI z-score was 2.64 ± 0.47. Mean BMI % of the 95th percentile was 147.98 ± 24.85. One-hundred sixty-nine out of 376 (45%) patients had measurement of vitamin D and lipids on same day as measurement of weight and height. The mean for number of days between the BMI measurement and measurement of 25(OH)D and lipids was 21.24 days (SD = 57.89, range = 0–343). The majority of study subjects were non-Hispanic (80.3%) and ethnicity data was marked as unknown or was missing for 40 out of 376 (10%) patients. Whites comprised 71.5% of the study population. The mean 25(OH)D concentration was 25.23 ± 10.10 ng/mL and median concentration was 24 ng/mL (range = 6.0–84.0). Two-hundred sixty-three (70%) children had 25(OH)D < 30 ng/Ml, and 113 subjects (30%) had 25(OH)D < 20 ng/mL. One-hundred and nine (29%) samples were obtained during summer (June–August), 100 (27%) during fall (September–November), 94 (25%) during spring (March–May), and 73 (19%) during winter (December–February). The concentrations of 25(OH)D were negatively associated with BMI z-score (*p* = 0.004) using the linear regression model, adjusted for age and gender.

The concentration of 25(OH)D was positively correlated with HDL cholesterol after adjustment for age, sex, BMI metric (z-score or % of the 95th percentile) and season of blood draw (*r^2^* = 0.08, *r* = 0.22, *β* = 0.16, 95% CI = 0.05–0.27, *p* = 0.004). This relationship was confirmed in non-Hispanics and in Whites but could not be examined in Hispanic children and non-White children due to small sample size. Using multivariate regression analyses and after adjustment for age, sex and BMI z-score, each 10 ng/mL increase in 25(OH)D was associated with an increase in HDL cholesterol by 1.64 mg/dL (95% CI = 0.5–2.7 mg/dL). 

**Figure 1:**
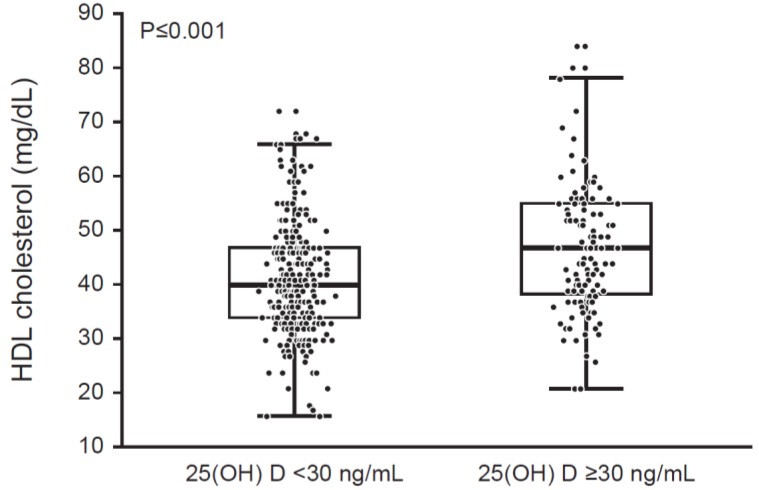
Box-plot showing differences in high-density lipoprotein (HDL) cholesterol between children with 25(OH)D < 30 ng/mL and those with 25(OH)D ≥ 30 ng/mL (*p* < 0.0001).

We then dichotomized the patients into two subgroups using 25(OH)D cut-off values of 30 ng/mL (recommended by the Endocrine Society, Washington, DC, USA) or 20 ng/mL (recommended by the Institute of Medicine, Washington, DC, USA). HDL cholesterol was lower in patients with 25(OH)D < 30 ng/mL in comparison to those with 25(OH)D ≥ 30 ng/mL (41.3 ± 10.2 vs. 46.4 ± 12 mg/dL, *p* < 0.0001) (see Table 2 and Figure 1). The prevalence of low HDL cholesterol (defined as < 40 mg/dL) was greater in children with 25(OH)D < 30 ng/mL compared to those with 25(OH)D ≥ 30 ng/mL (47.9% vs. 29.2%, OR 2.15 (1.33–3.51), *p* = 0.0019) (Table 3). The HDL cholesterol continued to be lower in patients with 25(OH)D < 30 ng/mL compared to those with 25(OH)D ≥ 30 ng/mL when we adjusted for season of blood draw (*p* < 0.001). Similarly, the prevalence of low HDL cholesterol continued to be significantly higher in patients with 25(OH)D < 30 ng/mL when we adjusted for season of blood draw (*p* = 0.002). There were no significant differences in HDL cholesterol or in proportion of patients with low HDL cholesterol between patients dichotomized into subgroups using a 25(OH)D cut-off of 20 ng/mL (*p* > 0.05 for both comparisons). The relationships between 25(OH)D and HDL cholesterol were not influenced by gender. 

We further stratified patients into three age groups (2–6 years, 7–11 years, and 12–18 years). Two-hundred sixty-three out of 376 (70%) patients were between 12 and 18 years of age, 82 (21.8%) were between 7–11 years, and only 31 (8.2%) were between 2 and 6 years of age. HDL cholesterol was significantly lower in patients children with 25(OH)D < 30 ng/mL relative to those with 25(OH)D ≥ 30 ng/mL in the two older age groups (7–11 years, *p* = 0.008 and 12–18 years, *p* = 0.008). There were no differences in HDL cholesterol between the two vitamin D subgroups (<30 ng/mL and ≥30 ng/mL) in the 2–6 years age group (*p* = 0.32). It is important to note that the number of subjects in the 2–6 years age group was very small (31 subjects, 8% of the total cohort) and therefore the statistical power to detect a difference in this age group was limited. 

There was no association between 25(OH)D concentration and total cholesterol or non-HDL cholesterol (*p* > 0.05) in the entire cohort or age stratified groups. Means of total cholesterol and non-HDL cholesterol were not different between children divided into sub-groups using cut-off 25(OH)D of 30 ng/mL (Table 2) or of 20 ng/mL. Additionally, the adjusted odds of elevated total cholesterol and elevated non-HDL cholesterol were not different between subgroups created using 25(OH)D cut-off of 30 ng/mL (Table 3, *p* > 0.05) or 20 ng/mL (*p* > 0.05). The relationship between 25(OH)D concentrations and total cholesterol and non-HDL cholesterol continued to remain not statistically significant after adjustment for season of blood draw.

## 4. Discussion

We found that low 25(OH)D concentration in children with severe obesity were associated with low HDL cholesterol which in turn has been associated with increased risk for cardiovascular disease. Specifically, children with 25(OH)D < 30 ng/mL had significantly greater odds of having low HDL cholesterol compared with children with 25(OH)D ≥ 30 ng/mL. To our knowledge, this is the largest study to examine the relationship between vitamin D status and lipids in children with severe obesity. We have also compared for the first time to our knowledge in this specific population, the 25(OH)D cut-off concentrations of both 20 ng/mL and 30 ng/mL in order to better understand a threshold 25(OH)D concentration that may be associated with greater odds of dyslipidemia. 

We found that every 10 ng/mL increment in 25(OH)D was associated with an increase in HDL cholesterol by 1.6 mg/dL after adjusting for gender, BMI z-score and age. We found that the positive association between 25(OH)D concentration and HDL cholesterol was present in children even after adjustment of confounding variables including severity of obesity and season of draw. On subgroup analysis by age, we noted these associations were present in children between 7 and 18 years of age but were not present in younger children. These findings may be related to the very small number of children between 2 and 6 years of age (8%) in the study cohort. Our findings of a positive association between 25(OH)D concentration and HDL cholesterol in children with severe obesity are similar to those previously reported by Smotkin-Tangorra and colleagues in children with obesity (mean BMI = 32.2, mean age = 12.9 years) attending a pediatric endocrine clinic in Brooklyn, New York [33]. Our findings are however in contrast to another study in 80 postmenarchal adolescent females (53 African American and 23 Caucasian American) with severe obesity residing in Georgia, USA [35]. These differences are likely related to differences in geographic location, ethnic distribution and pubertal staging. Unlike the previous study that comprised of postmenarchal adolescent females, our study subjects were of a wider age range (2–18 years), pubertal staging, and comprised of almost equal number of males and females. Positive association between 25(OH)D and HDL cholesterol concentrations has also been reported in normal weight children and children with lesser degree of overweight/obesity [26,27,28,31]. 

We found lower HDL cholesterol and greater prevalence of abnormal HDL cholesterol in children with 25(OH)D < 30 ng/mL relative to those with 25(OH)D ≥ 30 ng/mL. Similar findings with the 25(OH)D cut-off of 30 ng/mL were noted in children and young adults between ages 1–21 years from the National Health and Nutrition Examination Survey 2001–2004 [15]. However, we did not find differences in HDL cholesterol in sub-groups defined using the 25(OH)D threshold of 20 ng/mL, a level considered as optimal by some expert groups [19]. These findings were surprising given the significant correlations between 25(OH)D and HDL cholesterol in the entire cohort and the significant differences in HDL cholesterol seen using a cut-off of 30 ng/mL. Concentrations of 25(OH)D < 10 ng/mL have also been found to be associated with lower HDL cholesterol levels [33]. Presence of significant heterogeneity in the confounding variables such as pubertal stage, dietary habits, and physical activity may account for these findings and these results need to be validated in future studies. Our findings of the positive association between 25(OH)D concentrations and HDL cholesterol have significant public health importance given the increasing prevalence of severe obesity in children despite a recent plateau in the overall prevalence of childhood obesity [1]. A large proportion of children with severe obesity have multiple risk factors for cardiovascular disease including low HDL cholesterol and high blood pressure [6,7,8]. A large proportion of children with severe obesity also have suboptimal vitamin D status thereby increasing the potential implications of our findings [16,17]. The concentration of HDL cholesterol has been shown to be an independent inverse predictor of the risk of having an atherosclerotic cardiovascular disease [42], even when LDL cholesterol levels have been decreased as a result of treatment with a statin [43]. There appears to be 2–3% decrease in coronary artery disease risk with 1 mg/dL increment in HDL cholesterol [44]. In addition, increase in HDL by intravenous infusions of HDL leads to regression of established aortic fatty streaks and lipid deposits in cholesterol fed animals [45]. Our findings of lack of association between 25(OH)D concentrations and total cholesterol are similar to previous reports [33,36]. Total cholesterol was however noted to be higher in Swedish children with obesity in the presence of 25(OH)D < 15 ng/mL [34]. Conversely, low density lipoprotein cholesterol levels were noted to be positively associated with 25(OH)D concentrations in obese post menarchal adolescent females residing in Georgia [35]. These differences again may be related to differences in geographical location, age distribution, pubertal stage, race and ethnicity. 

The positive association between 25(OH)D concentration and HDL cholesterol highlights the need to examine the effect of increasing 25(OH)D by vitamin D supplementation on HDL cholesterol and potentially modify risk for cardiovascular disease. Our findings also highlight the importance of studying effect of other interventions that can increase 25(OH)D concentration such as increasing dietary intake of dairy products on risk factors for cardiovascular disease including low HDL cholesterol in children with severe obesity. 

Data on effect of vitamin D supplementation on lipids in children with severe obesity is sparse and further studies are warranted. Results from placebo controlled trials in children have given inconsistent results [46,47]. In one of our previous studies in obese adolescents, no change in HDL cholesterol was noted following 12 weeks supplementation with vitamin D3 2000 IU per day [46]. The dose of vitamin D3 was low and resulted in an increment of only 6 ng/mL in 25(OH)D concentrations. In contrast, increase in HDL cholesterol was noted in healthy 10–14 year old Iranian children who received 1000 IU vitamin D daily for one month [47]. The exact mechanism of positive association of 25(OH)D levels and HDL cholesterol is unclear. Vitamin D may influence HDL cholesterol via several mechanisms including effect on apolipoprotein A-1 production [48,49] or effect on cholesterol turnover or transport. Levels of 25(OH)D have been shown to be positively associated with levels of apolipoprotein A1, the major lipoprotein of HDL cholesterol in Belgian men and women [48]. 

The major strength of our study is the large sample size of children with severe obesity, a group with extremely high prevalence of dyslipidemia and vitamin D deficiency and, more importantly, at high risk for persistence of severe obesity into adulthood and development of CVD. Since 25(OH)D concentrations are routinely measured in our clinic for children with severe obesity, there was no selection bias with inclusion of only those patients that had additional risk factors for vitamin D deficiency. The large sample size allowed us to perform analyses using proposed threshold concentrations of both 20 ng/mL and 30 ng/mL. Another important strength was the use of a well validated LC-MS/MS assay for measuring 25(OH)D in a certified laboratory that is a referral center for vitamin D assays. 

A major limitation of our study is the cross-sectional design. Our findings reflect an association and do not imply causation. We were not able to look into the role of dietary patterns that influence 25(OH)D concentrations and lipids and can contribute to low 25(OH)D (particularly in winter) and dyslipidemia. Since the majority of children in our study were non-Hispanic and Whites, we were not able to examine the association between vitamin D status and lipids in Hispanics and non-Whites. We were also not able to adjust for the role of other potential confounders such as physical activity, skin color, sun exposure, pubertal staging, and parathyroid hormone levels, all of which can influence vitamin D status and/or lipids. The unequal distribution of patients in the two groups (25(OH)D ≥ 30 ng/mL and < 30 ng/mL) was a limitation with two thirds of our patients having 25(OH)D < 30 ng/mL. Lack of data on seasonal variation of 25(OH)D concentrations, and absence of a normal weight control group were other limitations. 

In summary, 25(OH)D concentrations in children with severe obesity were positively associated with HDL cholesterol levels. Our findings need to be validated in future studies that take into consideration ethnicity, pubertal development, and dietary habits. Additionally, prospective randomized controlled trials are warranted to determine if vitamin D supplementation or other interventions, such as modifications in diet or physical activity habits that increase 25(OH)D, can improve HDL cholesterol levels and potentially decrease cardiovascular risk among children with severe obesity. 

## Figures and Tables

**Table 1:** Demographic and laboratory characteristics of study subjects.

	Mean	Standard Deviation
Age (years)	13.48	3.95
Height (cm)	158.70	19.72
Weight (kg)	100.73	37.12
BMI (kg/m^2^)	38.32	8.12
BMI z-score	2.64	0.47
BMI % of the 95th percentile	147.98	24.85
25(OH)D (ng/mL)	25.2	10.1
Total cholesterol (mg/dL)	162.2	32.9
HDL cholesterol (mg/dL)	42.9	11.1
Non-HDL cholesterol (mg/dL)	119.3	32.7

Male: 168 (44.7%); Female: 208 (55.3%). 25(OH)D: 25-hydroxy vitamin D. BMI: body mass index. HDL: high-density lipoprotein.

**Table 2:** Laboratory characteristics in subjects with 25(OH)D < 30 ng/mL and in those with 25(OH)D levels ≥ 30 ng/mL.

	25(OH)D < 30 ng/mL (*n* = 263) ^a^	25(OH)D ≥ 30 ng/mL (*n* = 113) ^a^	*p*-Value
Age (years)	13.72 (3.64)	12.92 (4.54)	0.02 ^b^
BMI z-score	2.65 (0.45)	2.61 (0.53)	0.11 ^c^
BMI % of the 95th percentile	150.6 (26.4)	141.9 (19.6)	0.001 ^c^
Total cholesterol (mg/dL)	160.6 (31.4)	166 (36.1)	0.09 ^d^
HDL cholesterol (mg/dL)	41.1 (10.2)	47 (12)	<0.0001 ^d^
Non-HDL cholesterol (mg/dL)	119.5 (31.3)	119 (35.8)	0.89 ^d^

^a^: Data are presented as mean (standard deviation). ^b^: Adjusted for gender, BMI z-score; ^c^: Adjusted for gender, age; ^d^: Adjusted for gender, age, BMI metric (z-score or % of the 95th percentile).

**Table 3:** Prevalence of abnormal lipids in patients with 25(OH)D < 30 ng/mL and in those with 25(OH)D levels ≥ 30 ng/mL.

	25(OH)D < 30 ng/mL *n* (%)	25(OH)D ≥ 30 ng/dL *n* (%)	OR ^a^ (95% CI) ^b^	*p*-Value ^a^
High total cholesterol (≥200 mg/dL)	29 (11.03)	19 (16.8)	0.62 (0.33–1.29)	0.14
Low HDL cholesterol (<40 mg/dL)	126 (47.9)	33 (29.2)	2.15 (1.33–3.51)	0.0019
High non-HDL cholesterol (≥145 mg/dL)	53 (20.15)	28 (24.78)	0.74 (0.43–1.27)	0.26

^a^: Adjusted for gender, age and BMI metric (z-score or % of the 95th percentile). ^b^: Reference is 25(OH)D ≥30 ng/mL.
